# The Civil Hospitals and Dispensaries in the Madras Presidency

**Published:** 1901-09-21

**Authors:** 


### THE INSTITUTIONAL WORKSHOP.

### THE CIVIL HOSPITALS AND DISPENSARIES IN THE MADRAS PRESIDENCY.

The total number of institutions for the relief of the sick in the Madras Presidency at the end of 1899 was 523, of which 239 were hospitals and 284 dispensaries. These were of three classes, those which are maintained by (1) the State for the use of the public, the police, or the railway employees, (2) by local funds or municipal support, and (3) by private assistance. Medical relief was afforded to 4,226,308 persons, of which by far the largest proportion was men, the number of women being only 903,456 against 2,141,616 men. The Local Fund Dispensary at Venkatagirikotai was closed throughout the year, whilst 27 other institutions were not opened for broken periods amounting to 2,347 days in all, or an average of 87 days each. Six of these were dispensaries for women and children, and with two exceptions ceased to work, because no female medical practitioners were available. With one exception, all the others were closed upon the transfer of the medical subordinates in charge for plague duty elsewhere. As there seems little prospect of the plague dying out, at any rate for the present, unless the supply of medical officers can be increased, the same state of affairs is likely to arise each year. Hindus formed by far the largest class of patients, over 3,000,000 having come under treatment; Mussulmans coming next, but more than 8,000 Europeans benefited during the year from hospital treatment. The total number of in-patients was 46,510, of whom 65 per cent. were cured, 18 per cent. relieved, and 7 per cent. discharged otherwise.

The death-rate, though registered as 5 per cent., includes the large number of cases admitted moribund; therefore it would really be more correct, as far as hospital statistics are concerned, if it were placed at 4 per cent. Of the 66 deaths from septic diseases 60 of the patients were admitted in that condition, and only six became septic in hospital. The out-patients numbered 4,179,798, and it is satisfactory to be able to record that of these only 9 per cent. were "represented by friends," showing that the decided efforts made by the authorities to discourage this mode of gaining medical assistance is making way. There was a decrease in the number of cases of cholera and malarial fever admitted, and also a great fall in the total of patients coming to be treated for poisons or poisoned wounds. Also the total of eye affections was less. The results of the surgical operations were distinctly gratifying. Out of 120,000 patients operated upon 115,000 were cured and only 173 died: these, too, were all major and important operations. The midwives attached to the various institutions conducted 25,538 cases of labour, and the number is a distinct increase over that of the previous year. The income of the civil medical institutions was 10,93,766 rupees, and of this the Government contributed nearly $12\frac{1}{2}$ per cent.

